# Practical Points

**Published:** 1895-04-06

**Authors:** 


### PRACTICAL POINTS.

[Questions are invited for this column on any point in the administration of hospitals and asylums which may prove of difficulty or interest to our readers. All communications should be marked "Practical Points," addressed to the Editor of The Hospital, 428, Strand, W.C.]

**The Cost of Patients in Fever Hospitals.**—A Matron writes: *I am matron of a fever hospital containing 35 beds. During the whole time I have been in office I had 32 patients. I keep the books required by the Local Government Board, and I have worked very hard to make the place a success. I have made out the total expenditure for a year, with the sum spent per week on staff and patients severally, with this result: For the staff, which includes a porter, but no resident doctors, average per week, including beer,* 7s. 4d.; *per patient, mostly children, including wine and spirits,* 6s. 10d. *Half the patients have been diphtheria, half scarlet fever. Have I been extravagant as has been suggested? So far (we live four miles from our tradesmen), after the low diet stage we all eat exactly alike, nothing being wasted. Have you any diet sheets for infectious hospitals for staff and patients? You know more about the administration of hospitals than anyone else, and if you would kindly give me some decided and decisive views I should be very grateful. Unfortunately, all my "Hospitals" go into the wards to the nurses, and so I am not able to refer back to see if I could have helped myself without troubling you. If you can send by return any books or papers on the subject I shall be very glad.*

Seeing that it costs from 7s. to 9s. 6d. per week to maintain a lunatic in an asylum under the cheapest of conditions conceivable, where the cost can be cut down to the lowest possible dimensions, we are surprised and regret to hear that anyone charged with the duty of managing an infectious hospital should consider an average expenditure per head per week, including beer, of 7s. 4d. extravagant. So far from being extravagant, the ratepayers ought to know that the price is too little, if the best interests of the patients be considered, and that no publicly-managed infectious hospital attempts to reduce expenditure upon these cases, because the recovery of the patients is often materially quickened by a due regard to diet and comfort, both of which items often entail special expense. We find that the average cost per week per patient in the London Fever Hospital is about £1 17s. 6d., and the average cost at the Cork Street Fever Hospital, Dublin, about £1 5s. Any expenditure, in our view, upon fever cases in a hospital like yours, which is less than £1, is justifiable, if it is not even necessary. Complaints continually reach us of the inadequate provision made at the public fever hospitals for the comforts, the nursing, the diet, and the care of the patients. Fever hospitals should not be a mockery, but a sustenance and stay to the population who reside in the district where they are situated.

**The Number of the Staff in Small Hospitals.**—A Correspondent writes: *I shall deem it a great favour if you will kindly give your opinion as to whether our staff is proportionate to our institution. We can make up forty beds. During the last year* (1894) 197 *in-patients were treated in the wards, and about* 2,200 *out-patients were also under treatment. Our average number of occupied beds is* 16; *the highest number occupied at one time being* 30, *and the lowest nine. Our staff consists of a house surgeon, lady superintendent, head nurse, and two probationers, one of whom is constantly on night duty, and the domestic staff is made up of cook, kitchen-maid, housemaid, laundrymaid, and porter; the latter lives and takes his meals at home, and his duties consist mainly of looking after the grounds, carrying coals, and cleaning outside windows, &c. He does not answer the door or assist in any way, except on emergency, with the patients. I might add that as we are the centre of a large district comprising the greater part of the county, it is necessary for patients to have all their personal washing done in the house, in addition to our usual work. The patients pay six shillings a week towards cost of maintenance and washing.*

The question as to whether a particular staff is proportionate to the needs of a particular institution depends upon many considerations. In the above institution, for example, it must be manifest that one laundrymaid is certainly not sufficient to handle all the personal washing of thirty or even sixteen patients, in addition to that of the staff. It is further manifest that one head nurse and one probationer could not properly attend to thirty patients during the day, occupying several wards in the same institution. Even sixteen patients in such circumstances, when the cases are severe, is more than a trained nurse and her assistant ought to have charge of. No committee ought to take the responsibility of leaving thirty patients, many of them in a highly critical state, in charge of one probationer during the night. If the acute cases and cases of accident are so numerous and important as to necessitate the appointment of a house surgeon, it follows that there should be at least four probationers and two head nurses to do the work of this institution. The fact that each patient pays six shillings a week towards cost of maintenance, emphasises the importance and justice of the re-arrangement of the staff, and an increase to its numbers. We shall hope to hear that the necessary changes here indicated have been carried out without delay or hesitation.

